# Gallstones, cholecystectomy, and the risk for developing pancreatic cancer

**DOI:** 10.1038/sj.bjc.6600193

**Published:** 2002-04-08

**Authors:** E S Schernhammer, D S Michaud, M F Leitzmann, E Giovannucci, G A Colditz, C S Fuchs

**Affiliations:** Channing Laboratory, Department of Medicine, Brigham and Women's Hospital and Harvard Medical School, Boston, Massachusetts, USA; Ludwig Boltzmann-Institute for Applied Cancer Research, KFJ-Spital, Vienna, Austria; National Cancer Institute, Nutritional Epidemiology Branch, 6120 Executive Blvd., Rockville, MD 20852, Maryland, USA; Department of Nutrition, Harvard School of Public Health, 665 Huntington Ave., Boston, MA 02115, Massachusetts, USA; Department of Epidemiology, Harvard School of Public Health, Boston, Massachusetts, USA; Harvard Center for Cancer Prevention, Boston, Massachusetts, USA; Epidemiology Program, Dana Faber/Harvard Cancer Center, Boston, Massachusetts, USA; Department of Adult Oncology, Dana-Farber Cancer Institute, 44 Binney Street, Boston, MA 02115, Massachusetts, USA

**Keywords:** pancreatic cancer, gallstones, cholecystectomy, risk factors, prospective cohort study

## Abstract

We examined the relation between gallstones, cholecystectomy, and the development of pancreatic cancer in the Nurses' Health Study and the Health Professionals Follow-up Study. Among 104 856 women and 48 928 men without cancer at baseline, we documented 349 cases of pancreatic cancer during up to 16 years of follow-up. Participants were classified according to a history of gallstones or cholecystectomy. The age-adjusted relative risk of pancreatic cancer following cholecystectomy or diagnosis of gallstones was 1.31 (95% CI, 0.93–1.83). However, adjustment for other pancreatic cancer risk factors attenuated the association (RR=1.11, 95% CI, 0.78–1.56); this risk did not increase with increasing time following cholecystectomy or gallstones. Gallstones or cholecystectomy do not appear to be significant risk factors for pancreatic cancer.

*British Journal of Cancer* (2002) **86**, 1081–1084. DOI: 10.1038/sj/bjc/6600193 www.bjcancer.com

© 2002 Cancer Research UK

## 

Pancreatic cancer is the fifth leading cause of cancer-related mortality in the US (Greenlee *et al*, 2001). New environmental risk factors have consistently been linked to the risk of pancreatic cancer. Animal models suggest an important role for cytokines in experimental pancreatic carcinogenesis (Watanapa and Williamson, 1993). Cholecystokinin (CCK) has been shown to stimulate both gallbladder contraction and pancreatic enzyme secretion (Marx *et al*, 1987; Smith *et al*, 1990) and to have a trophic effect on pancreatic acinar cells (Rivard *et al*, 1991). The presumed increased release of CCK following cholecystectomy (Hyvarinen and Partanen, 1987) might contribute to an increased risk for pancreatic cancer risk following gallbladder removal. In addition, cholecystectomy may suppress the normal inhibitory effect of CCK on the Sphincter of Oddi (Luman *et al*, 1997). The presence of gallstones, on the other hand, appears to be associated with chronic pancreatitis (Hardt *et al*, 2001), but whether chronic pancreatitis predisposes to pancreas cancer remains controversial (Lowenfels *et al*, 1993; Ekbom *et al*, 1994). Some of the earlier studies reported relative risks of pancreatic cancer of 1.2–2.0 among individuals who had undergone a cholecystectomy previously (Haines *et al*, 1982; Shibata *et al*, 1994; Ekbom *et al*, 1996; Chow *et al*, 1999; Gullo, 1999; Silverman *et al*, 1999; Coughlin *et al*, 2000); however, the strength of the association remains uncertain because of the retrospective design of most analyses and the reliance on next-of-kin respondents. Prospective studies can overcome these limitations. We examined the relation between cholecystectomy or gallstones and pancreatic cancer risk in two large cohorts, the Nurses' Health Study (NHS) and the Health Professionals Follow-up Study (HPFS).

## MATERIALS AND METHODS

### Study cohorts

The Nurses' Health Study (NHS) enrolled 121 700 female, registered nurses aged 30–55 years in 1976, and information on their health status, medical history, and known and suspected risk factors for cancer and coronary heart disease was gathered through mailed questionnaires. The Health Professionals Follow-up Study (HPFS) began in 1986, when 51 529 American male dentists, optometrists, osteopaths, pharmacists, podiatrists, and veterinarians aged 40–75 years completed a mailed questionnaire on health-related questions similar to those in NHS. Follow-up questionnaires were sent biennially to participants in both cohorts.

### Ascertainment of cholecystectomy or gallstone diagnosis

In 1982, the study participants of the NHS were asked about their history of cholecystectomy and the year of surgery, with information updated biennially. History of gallstones that were not removed was also assessed on the 1982 questionnaire but was updated only through 1986. In the HPFS, participants were asked about their history of cholecystectomy or gallstones at baseline, and both questions were updated with all subsequent questionnaires.

### Documentation of pancreatic cancer and deaths

We included confirmed pancreatic cancers diagnosed between the return of the 1982 questionnaire and June 1, 1998, for the NHS and between the return of the 1986 questionnaire and January 1, 1998, for the HPFS. With permission from study participants, we confirmed pancreatic cancer through physicians' review of their medical records. If permission was denied, we attempted to confirm the self-reported cancer with an additional letter or phone call. We also searched the National Death Index to identify deaths among the nonrespondents to each 2-year questionnaire. The computerised National Death Index is a highly sensitive method for identifying death in this cohort (Stampfer *et al*, 1984). For all deaths attributable to pancreatic cancer, we requested permission from family members (subject to state regulation) to review the medical records. Pancreatic cancer was considered the cause of death if the medical records or autopsy report confirmed fatal pancreatic cancer or when pancreatic cancer was listed as the underlying cause of death without another, more plausible cause. In this analysis, we included 170 cases of pancreatic cancer in the women and 127 in the men.

### Statistical analysis

We excluded participants who did not answer the baseline questionnaire on gallstones and cholecystectomy (1982 in the NHS; 1986 in the HPFS) and all participants who reported a history of cancer (with the exception of non-melanoma skin cancer). Since the diagnosis of gallstones or the need for cholecystectomy might have been triggered by early symptoms associated with pancreatic cancer, we also excluded the first 2 years of follow-up for all study participants after their first report of either cholelithiasis or cholecystectomy. Therefore, for NHS participants who did not report gallstones or cholecystectomy between 1984 and 1986, we initiated follow-up with the date of return of the baseline 1982 questionnaire, whereas, for women who reported gallstones or cholecystectomy between 1984 and 1986, follow up began in 1988. In the HPFS, however, the baseline 1986 questionnaire assessed history of gallstones and cholecystectomy in timeframes that were larger than a 2-year interval. There, for all HPFS participants, we initiated follow-up with the date of return of the 1988 to ensure that persons reporting a history of either gallstones or a cholecystectomy were excluded from the succeeding 2 years of analysis only. In both cohorts, we computed person years of follow-up to the date of diagnosis of pancreatic cancer, death from any cause, or to the end of the study period (June 1, 1998, for women, and February 1, 1998 for men) whichever occurred first. After these exclusions, 104 856 women and 48 928 men were eligible for follow-up, and 1 405 681 person years were accrued in the NHS and 441 387 in the HPFS.

The primary analysis used incidence rates with person-years of follow-up in the denominator. We used relative risk (RR) as the measure of association; RR was defined as the incidence rate of pancreatic cancer among participants with a history of gallstones or a cholecystectomy divided by the incidence rate among participants without such a history. In the NHS, person time with gallstones history from 1986 on was based on the cumulative history of gallstones up to 1986. For each cohort, we used Cox proportional hazards models to adjust for other potential risk factors for pancreatic cancer. Although rapid weight loss in obese patients constitutes a risk factor for gallstones (Yang *et al*, 1992), pancreatic cancer is also frequently associated with profound weight loss. Therefore, we did not adjust for weight change or most recent body mass index but used baseline body mass index (1976 for the NHS, 1986 for the HPFS), which appeared to be the most important weight risk factor for pancreatic cancer in these cohorts (Michaud *et al*, 2001). We used a random-effects model for the log of the RR (DerSimonian and Laird, 1986) to pool the data from the two cohorts.

## RESULTS

A total of 206 women and 143 men were diagnosed with pancreatic cancer during 2 162 077 person-years of follow-up. Of these 349 persons, 333 (96%) died of the disease. At baseline, 9.2% of the women (16.4% by 1996) and 3.7% of the men (7.1% by 1996) reported gallstones or a history of cholecystectomy. Characteristics of the study population are described in Table 1

**Table 1:**
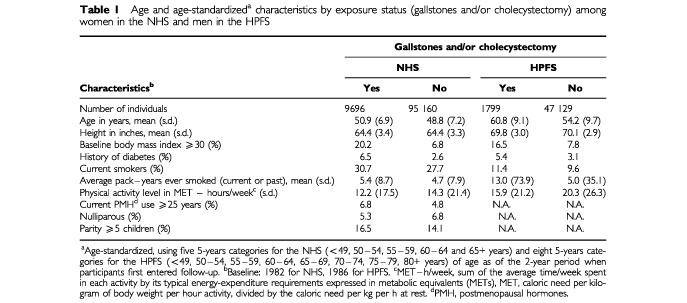
Age and age-standardized^a^ characteristics by exposure status (gallstones and/or cholecystectomy) among women in the NHS and men in the HPFS

.

In our primary analysis we excluded participants who reported a history of cholecystectomy or gallstones from the proceeding 2 years of analysis, which left 170 women and 127 men with a diagnosis of pancreatic cancer. Of these, women with a history of gallstones or a cholecystectomy experienced a modest elevation in risk for pancreatic cancer (age-adjusted RR=1.47, 95% CI, 1.00–2.16), whereas men with such a history did not experience an obvious increase in risk (Table 2

**Table 2:**
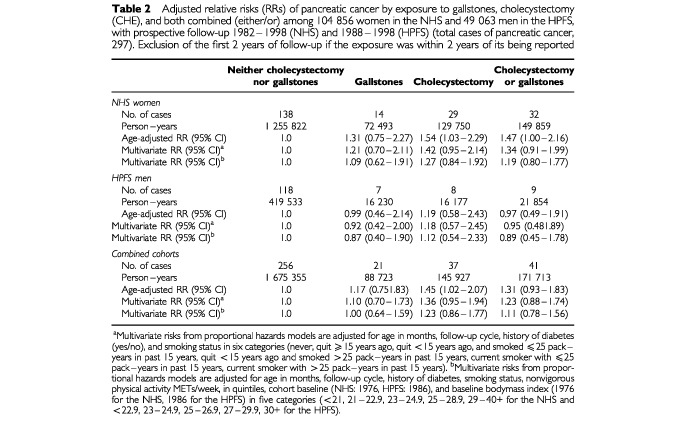
Adjusted relative risks (RRs) of pancreatic cancer by exposure to gallstones, cholecystectomy (CHE), and both combined (either/or) among 104 856 women in the NHS and 49 063 men in the HPFS, with prospective follow-up 1982–1998 (NHS) and 1988–1998 (HPFS) (total cases of pancreatic cancer, 297). Exclusion of the first 2 years of follow-up if the exposure was within 2 years of its being reported

). Among men and women combined, the age-adjusted RR was 1.31 (95% CI, 0.93–1.83). However, after adjusting for other factors, including body mass index and physical activity, the RR was 1.11 (95% CI, 0.78–1.56). Adjustment for postmenopausal hormone use and parity among women did not substantially alter our estimates (data not shown).

Multivariate RRs in the analyses that included all 349 diagnosed pancreatic cancer case subjects were not substantially different from the primary analyses for women (RR=1.16, 95% CI, 0.81–1.66), men (RR=0.96, 95% CI, 0.52–1.75), or women and men combined (RR=1.10, 95% CI, 0.81–1.50).

We examined the relation between time since cholecystectomy and risk of pancreatic cancer. Among individuals with a cholecystectomy 10 or fewer years in the past, the multivariate RR for pancreatic cancer was 1.12 (95% CI, 0.66–1.89), whereas for more than 10 years in the past, the RR was 1.13 (95% CI, 0.76–1.68). For cholecystectomy more than 20 years in the past, the RR was 0.88 (95% CI, 0.49–1.59). These findings were not materially different when we examined time interval since cholecystectomy or a diagnosis of gallstones; for individuals with a history of either cholecystectomy or gallstones more than 10 years previously, the RR for pancreatic cancer was 1.16 (95% CI, 0.79–1.70).

## DISCUSSION

In two prospective cohorts of women and men, risk for pancreatic cancer following diagnosis of gallstones or cholecystectomy was modestly but not significantly increased. This risk was attenuated substantially after adjustment for potential confounders, including body mass index and physical activity. Furthermore, the risk did not increase in the decades following either cholecystectomy or diagnosis of gallstones.

The prospective nature of our studies precluded recall bias and the need of next-of-kin respondents. Moreover, to avoid misclassification of exposure, we updated reports of cholecystectomy and gallstones biennially. Because identification of deaths is highly accurate in these cohorts (Stampfer *et al*, 1984), differential follow-up is unlikely. Nonetheless, case number may have compromised the ability to explore further details in these cohorts. Based on the available statistical power, we cannot completely exclude an effect for cholecystectomy or cholelithiasis, although in both cohorts the effect appears to be consistently small. It is possible that gallstones and pancreatic cancer have similar etiologic factors (e.g. hyperinsulinemia), which may account for an association between the two. Controlling for physical activity and BMI, a strong determinant of hyperinsulinaemia, reduced the modest association between cholecystectomy and pancreatic cancer.

Previous studies have investigated a potential association between gallstones or cholecystectomy and risk of pancreatic cancer. Results from case-control studies have been conflicting (Haines *et al*, 1982; Shibata *et al*, 1994; Ekbom *et al*, 1996; Coughlin *et al*, 2000), although some indicate a 30–70% increased risk of pancreatic cancer in association with gallstones or after a cholecystectomy (Chow *et al*, 1999; Gullo, 1999; Silverman *et al*, 1999; Silverman, 2001). In the largest case-control study, Silverman (2001) observed a RR of 1.7 (95%CI, 1.0–1.3). One prospective study found an increased RR for pancreatic cancer of 1.99 (95% CI, 1.08–3.68) associated with cholecystectomy after adjustment for sex, age, and cigarette smoking (Shibata *et al*, 1994). However, that study did not adjust for potential confounders such as obesity and was limited by a small number of cases. The only other cohort study observed a slightly elevated risk for pancreatic cancer in men with a history of gallstones (RR 1.2, 95% CI, 1.0–1.5), but their ability to control for potential confounding factors in this large cohort was compromised by incomplete follow-up (Coughlin *et al*, 2000). Few studies have examined the time interval between cholecystectomy and incidence of pancreatic cancer. Silverman (2001) found a 70% excess risk of pancreatic cancer 20 or more years after a cholecystectomy. In contrast, Hyvarinen and Partanen found a statistically significant increased risk of pancreatic cancer within 5 years after cholecystectomy (*P*=0.007) but not thereafter (Hyvarinen and Partanen, 1987). Moreover, Ekbom *et al* (1996) found no increase in risk with increased duration after cholecystectomy.

Although the age-adjusted findings in our two cohorts are somewhat more consistent with previous positive results, multivariate adjustment suggests considerable confounding in this relation.

In summary, our findings do not support a strong relation between gallstones or prior cholecystectomy and pancreatic cancer risk. The effect of gallstones and cholecystectomy on pancreatic cancer risk appears small and residual confounding by risk factors for both cholecystectomy and pancreatic cancer may have explained the results in previous studies.
